
JOURNAL INFORMATION
==============================
NLM Title Abbreviation: Biospektrum (Heidelb)
Journal ID: Biospektrum (Heidelb)
NLM Journal ID: 9615685

Biospektrum

ISSN: 0947-0867
EISSN: 1868-6249

ARTICLE INFORMATION
==============================
PMCID: PMC8233640
PMID: 34219980
DOI: 10.1007/s12268-021-1600-x
Article ID: 1600
Article version: 1
Subjects: Wissenschaft

mRNA-Strukturen steuern die posttranskriptionelle Genregulation

Lichtenthaeler Chiara
Oberstrass Lasse
Weigand Julia E. julia.weigand@tu-darmstadt.de

grid.6546.1 0000 0001 0940 1669 Fachbereich Biologie, Technische Universität Darmstadt, Schnittspahnstraße 10, D-64287 Darmstadt, Deutschland
Electronic publication date: 2021 Jun 26
Print publication date: 2021
Volume: 27
Issue: 4
First page: 351
Last page: 354
Copyright: © Die Autoren 2021
License: Open Access: Dieser Artikel wird unter der Creative Commons Namensnennung 4.0 International Lizenz veröffentlicht, welche die Nutzung, Vervielfältigung, Bearbeitung, Verbreitung und Wiedergabe in jeglichem Medium und Format erlaubt, sofern Sie den/die ursprünglichen Autor(en) und die Quelle ordnungsgemäß nennen, einen Link zur Creative Commons Lizenz beifügen und angeben, ob Änderungen vorgenommen wurden. Die in diesem Artikel enthaltenen Bilder und sonstiges Drittmaterial unterliegen ebenfalls der genannten Creative Commons Lizenz, sofern sich aus der Abbildungslegende nichts anderes ergibt. Sofern das betreffende Material nicht unter der genannten Creative Commons Lizenz steht und die betreffende Handlung nicht nach gesetzlichen Vorschriften erlaubt ist, ist für die oben aufgeführten Weiterverwendungen des Materials die Einwilligung des jeweiligen Rechteinhabers einzuholen. Weitere Details zur Lizenz entnehmen Sie bitte der Lizenzinformation auf http://creativecommons.org/licenses/by/4.0/deed.de.
License URL: https://creativecommons.org/licenses/by/4.0/

issue-copyright-statement: © Springer-Verlag GmbH Deutschland, ein Teil von Springer Nature 2021

==============================
Posttranscriptional regulation at mRNA level is defined not only by sequence, but also by the structure of the mRNA. Structures in the untranslated regions control stability, translation and localization of an mRNA. Evolutionary conservation can be used to identify such regulatory active structures in mRNAs. For example, constitutive decay element (CDE) stem-loops are recognized by Roquin proteins in an exclusively shape-specific manner. Strikingly, some CDEs can serve a dual function in gene repression depending on their folding status.

■ RNA erfüllt viele verschiedene und essenzielle zelluläre Funktionen, die weit über ihre Rolle als passiver Träger der genetischen Information hinausgehen. Ähnlich wie Proteine können RNAs komplexe 3D-Strukturen annehmen. Zusätzlich kann eine RNA verschiedene Strukturen in Abhängigkeit der Bindung von Liganden einnehmen. Beispiele hierfür sind Ribozyme, Riboswitche und tRNAs.

Darüber hinaus ist die 3D-Struktur einer mRNA, unabhängig von der reinen linearen Sequenzinformation, an vielen regulatorischen Prozessen in der Zelle beteiligt. Ein aktuelles Beispiel ist das Genom von SARS-CoV-2, welches aus einer einzelsträngigen RNA besteht. Dieses besitzt distinkte, komplex gefaltete RNA-Strukturen, wie z. B. einen Pseudoknoten, der das Ribosom dazu veranlasst, von einem Leserahmen in den anderen zu wechseln [1].

Gerade in den untranslatierten Regionen (UTRs) finden sich viele solcher Strukturelemente, deren Wirkung oft durch die Interaktion zwischen RNA und RNA-bindenden Proteine (RBPs) bedingt ist. Strukturen in mRNAs beeinflussen das prä-mRNA-Spleißen, die zelluläre Lokalisierung, die Translationsrate sowie die mRNA-Stabilität. mRNA-Strukturen sind somit essenziell für die posttranskriptionelle Genregulation und erlauben so, das Proteom präzise an interne und externe Signale anzupassen. Beispiele für bekannte Strukturelemente, welche die mRNA-Funktion kontrollieren, umfassen z. B. *iron responsive elements*, welche die Eisenhomöostase regulieren, oder Zipcodes, welche die Lokalisation von mRNA-Molekülen in der Zelle steuern. Zudem ist die Halbwertszeit einer mRNA genauestens reguliert. Neben linearen Motiven, wie MREs (*microRNA response elements*) und AREs (AU-reiche Elemente), sind auch hier RNA-Strukturen von großer Bedeutung. Die *constitutive* und *alternative decay elements* (CDEs und ADEs), die aus einer einzelnen Haarnadelstruktur bestehen, werden von Roquin-Proteinen erkannt und regulieren den mRNA-Abbau (**Abb. 1**, [2]).

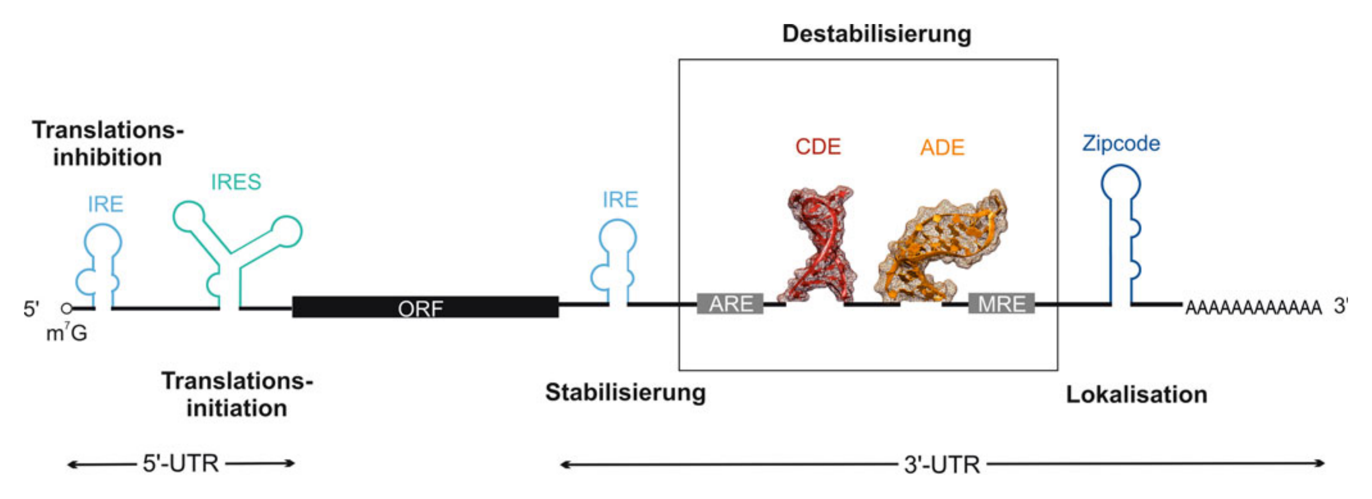

▲ **Abb. 1:** Regulatorische (Struktur-)Elemente und ihre Funktion. IRE: *iron responsive element*; IRES: interne ribosomale Eintrittsstelle; ORF*: open reading frame*; ARE: AU-reiches Element; MRE: *microRNA response element*; CDE: *constitutive decay element* (aus PDB ID 2n2o [8]); ADE: *alternative decay element* (aus PDB ID 5f5h [9]); UTR: untranslatierte Region.

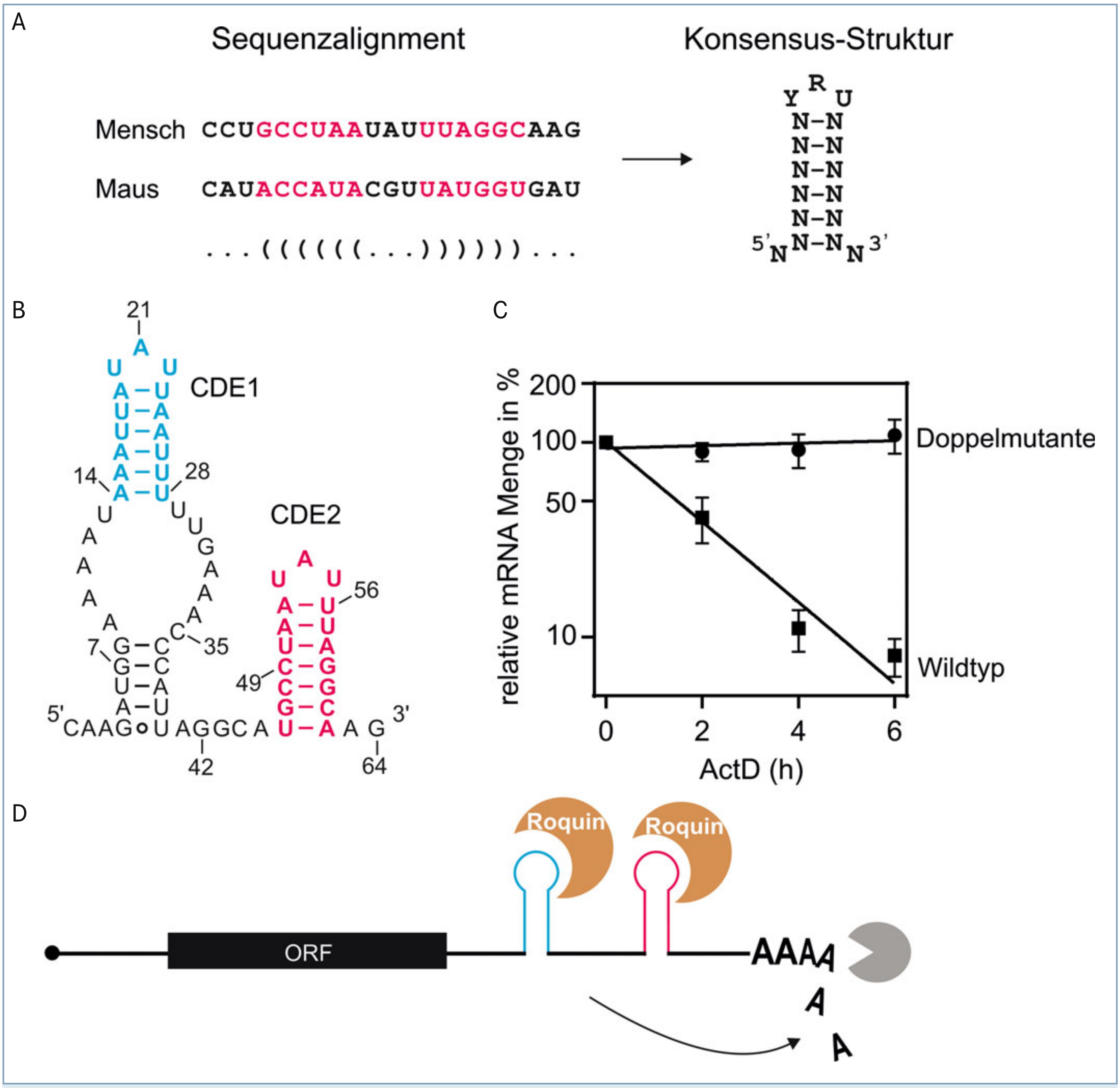

▲ **Abb. 2:** Die 3′-UTR von *UCP3* enthält zwei Roquin-Bindestellen [2]. **A**, Bioinformatische Vorhersage evolutionär konservierter RNA-Strukturen (N = A, U, G oder C; R = A oder G; Y = C oder U). **B**, *UCP3*-Tandem-CDE Motiv. **C**, Lebensdauer einer mRNA mit Wildtyp und mutiertem Tandem-CDE-Motiv. Durch die Behandlung der Zellen mit Actinomycin D (ActD) kommt es zu einem Transkriptionsstopp, wodurch die Lebensdauer einer mRNA bestimmt werden kann. **D**, Die Bindung von Roquin an Stammschleifen in der 3′-UTR führt zur Rekrutierung von Deadenylierungsfaktoren. Abbildung modifiziert nach [2], CC BY 4.0.

Oft sind solche mRNA-Strukturen evolutionär konserviert. Sie sind nicht von der exakten Sequenz abhängig, da verschiedene Abfolgen von Nukleotiden gleiche Strukturelemente ausbilden können. Konservierte mRNA-Strukturen sind daher nicht so leicht zu entdecken. Findet man konservierte Strukturen, so ist dies ein starker Hinweis auf deren Funktionalität.

### Von evolutionärer Strukturkonservierung hin zur Entdeckung neuartiger Regulationselemente

Durch den Vergleich aller UTRs von Mensch und Maus wurden evolutionär konservierte RNA-Strukturen genomweit vorhergesagt. Bei der Analyse wurde der Schwerpunkt auf sequenzunabhängige Strukturkonservierung gelegt (**Abb. 2A**). So identifizierten wir eine neuartige Roquin-Tandem-Bindestelle innerhalb der 3′-UTR der *UCP3*-mRNA (**Abb. 2B**). UCP3 ist ein mitochondriales Membranprotein, das mit der Entwicklung von Diabetes mellitus Typ II und Adipositas in Verbindung gebracht wird [3]. Mutationsanalysen in humanen Zelllinien bestätigten, dass zwei Stammschleifen, die beide einen identischen Triloop aufweisen, notwendig und ausreichend sind, um die Proteinproduktion zu inhibieren. Diese negative Regulation wird durch einen beschleunigten mRNA-Abbau hervorgerufen (**Abb. 2C**). Die identifizierten Stammschleifen aus der *UCP3*-mRNA ähneln CDEs, bekannten Roquin-Bindestellen. Die Bindung von Roquin an CDEs führt zur Rekrutierung von Deadenylierungsfaktoren, wodurch die mRNA destabilisiert und in Konsequenz abgebaut wird (**Abb. 2D**).

Das erste CDE wurde in der 3′-UTR der *TNF*(Tumornekrosefaktor)-mRNA TNF (Tumornekrosefaktor) beschrieben. Hier wurde durch Mutationsanalysen ein Konsensus bestimmt [4], welcher jedoch nicht mit anderen Roquin-Bindestellen wie dem *UCP3*-Tandem-CDE übereinstimmt. Zusätzlich zeigten Strukturbestimmungen von Roquin-CDE-Komplexen eine formspezifische Erkennung unabhängig von exakten Sequenzanforderungen [5]. Dies deutet auf eine vielseitigere Bindung von Roquin an Stammschleifen hin, welche wir durch Mutationsanalyse zeigen konnten.

## Die Art der Proteininteraktion ist abhängig vom Faltungsstatus der RNA

Obwohl sich ihre Sequenzen deutlich von den zuvor analysierten CDEs unterscheiden, zeigten die *UCP3*-CDEs, sowohl ungebunden als auch im Komplex mit Roquin, die bekannte CDE-Faltung (**Abb. 3A**). Somit bestätigen die Strukturdaten der *UCP3*-CDEs vollständig die Existenz eines sequenzunabhängigen Konsensus für CDEs.

Auffallend ist, dass beide CDEs AU-reich sind. Für AREs ist bekannt, dass sie mRNA-Abbau vermitteln. Somit könnten AU-reiche CDEs in linearer Form auch als AREs dienen (**Abb. 3B**). AREs sind die derzeit am besten untersuchten *cis*-regulatorischen Elemente, die die mRNA-Lebensdauer modulieren. Ursprünglich wurden sie in mRNAs entdeckt, die für *early response*-Gene codieren. Inzwischen sind sie als weit verbreitete *cis*-regulatorische Elemente anerkannt, die die Abundanz von mRNAs codierend für Wachstumsfaktoren, proinflammatorische Cytokine und Protoonkogene kontrollieren. Bislang wurden über 20 ARE-bindende Proteine identifiziert. Ein prominentes Beispiel, das den ARE-abhängigen mRNA-Abbau fördert, ist AUF1 [6].

Kommt es zur Faltung der CDEs in der 3′-UTR von *UCP3*, können diese Strukturen von Roquin gebunden werden. Die Bindung des Proteins führt zu einer Stabilisierung der mRNA-Struktur und verhindert so das Auffalten der doppelsträngigen RNA-Region. Liegt der Bereich der 3′-UTR linear vor, kann er von ARE-BPs (Bindeproteine) wie AUF1 gebunden werden (**Abb. 3B**). In der Tat konkurrieren Roquin und AUF1 um die Bindung an die AU-reichen CDEs, wie wir *in vitro* zeigen konnten (**Abb. 3C**, **D**). In diesen Fällen ist der Roquin-vermittelte vs. ARE-vermittelte mRNA-Abbau abhängig vom RNA-Faltungsstatus.

Zusammenfassend bestätigen die Strukturanalysen der AU-reichen *UCP3*-CDEs die sequenzunabhängige Bindung an CDEs. Weiterhin konnten wir zeigen, dass AU-reiche CDEs auch von ARE-BPs *in vitro* erkannt werden können und somit möglicherweise eine Doppelfunktion im mRNA-Abbau erfüllen.

## Fazit

Betrachtet man die Tatsache, dass (a) RNA Strukturen wandelbar sind und sich durch eine Umfaltung andere Interaktionsmöglichkeiten ergeben, (b) mehrere Bindestellen auf einer RNA überlappen können und (c) ein RBP oft mehrere RNA-Bindedomänen sowie Präferenz für verschiedene RNA-Motive besitzen kann, so wird die schiere Vielzahl an kooperativen sowie konkurrierenden Regulationsmöglichkeiten deutlich. Um das Verständnis posttranskriptioneller Genregulation weiter zu verbessern, muss daher in zukünftiger Forschung die Konkurrenz zwischen RBPs *in vivo* untersucht werden. Weiterhin ist es wichtig das Zusammenspiel einzelner RNA-Bindedomänen in einem RBP zu entschlüsseln. Dazu sollten Hochdurchsatzverfahren gekoppelt an *next generation sequencing* zum Einsatz kommen, mit denen schnell große Bibliotheken an RNA-Strukturen gleichzeitig untersucht werden können – zum einen in Hinsicht auf ihre Funktion *in vivo*, z. B. durch FACS-basierte *massive parallel reporter assays,* und zum anderen hinsichtlich der Erken-

Hier steht eine Anzeige.
Springer

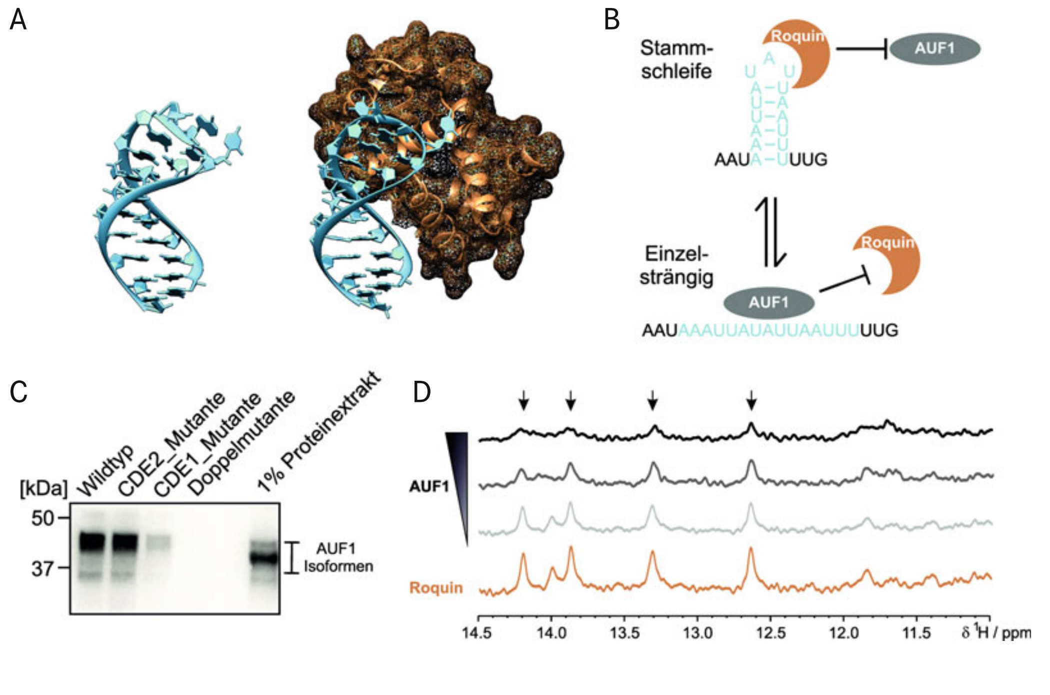

◀ **Abb. 3**: Strukturabhängige Kompetition um AU-reiche CDEs [10]. **A**, Struktur eines freien CDE im Vergleich zum Komplex mit der ROQ-Domäne von Roquin-1 (PDB IDs 6xwj, 6tqa). **B**, Roquin und AUF1 kompetitieren um die Bindung an CDE1. **C**, Immunoblot gegen AUF1 nach RNA-Affinitätsaufreinigung mit verschiedenen *UCP3*-RNAs. Durch Mutation von CDE1 geht die Bindung von AUF1 verloren. **D**, NMR-Analysen des CDE1-Roquin-Komplexes alleine und mit zunehmender AUF1 Konzentration zeigen, dass AUF1 mit Roquin um die RNA-Bindung kompetitiert. Die Signale, welche durch die Bindung von Roquin an CDE1 entstehen (Pfeile), werden durch AUF1 aufgelöst. Abbildung modifiziert nach [10], CC BY-NC 4.0.

nung durch RNA-bindende Domänen, z. B. durch RNA Bind-n-Seq [7].


Funding note: Open Access funding enabled and organized by Projekt DEAL.

Chiara Lichtenthaeler 2014–2017 Biowissenschaftenstudium an der Universität Frankfurt a. M. 2017–2020 Molekulare-Medizin-Studium an der Universität Frankfurt a. M. Seit 2020 Promotion am Fachbereich Biologie der TU Darmstadt im Labor von PD Dr. J. Weigand.

Lasse Oberstrass 2008–2012 Ausbildung als CTA. 2012–2018 Studium Biomolecular Engineering an der TU Darmstadt. Seit 2020 Promotion am Fachbereich Biologie der TU Darmstadt im Labor von PD Dr. J. Weigand.

Julia E. Weigand 2000–2005 Biologiestudium an der Universität Erlangen-Nürnberg. 2006–2009 Promotion im Labor von Prof. Dr. B. Suess an der Universität Erlangen-Nürnberg und der Universität Frankfurt a. M. 2010–2011 Postdoktorandin im Labor von Prof. Dr. S. Dimmeler an der Universität Frankfurt a. M. Seit 2012 Gruppenleiterin am Fachbereich Biologie der TU Darmstadt. 2020 Habilitation in Molekularbiologie, TU Darmstadt.


Literatur

[1] Wacker A Weigand JE Akabayov SR Secondary structure determination of conserved SARS-CoV-2 RNA elements by NMR spectroscopy Nucleic Acids Res 2020 48 12415 12435 10.1093/nar/gkaa1013 33167030 PMC7736788
[2] Braun J Fischer S Xu ZZ Identification of new high affinity targets for Roquin based on structural conservation Nucleic Acids Res 2018 46 12109 12125 10.1093/nar/gky908 30295819 PMC6294493
[3] Pohl EE Rupprecht A Macher G Important trends in UCP3 investigation Front Physiol 2019 10 470 10.3389/fphys.2019.00470 31133866 PMC6524716
[4] Leppek K Schott J Reitter S Roquin promotes constitutive mRNA decay via a conserved class of stem-loop recognition motifs Cell 2013 153 869 881 10.1016/j.cell.2013.04.016 23663784
[5] Schlundt A Heinz GA Janowski R Structural basis for RNA recognition in roquin-mediated post-transcriptional gene regulation Nat Struct Mol Biol 2014 21 671 678 10.1038/nsmb.2855 25026077
[6] von Roretz C Marco SD Mazroui R Turnover of AU-rich-containing mRNAs during stress: a matter of survival WIREs RNA 2011 2 336 347 10.1002/wrna.55 21957021
[7] van Nostrand EL Freese P Pratt GA A large-scale binding and functional map of human RNA-binding proteins Nature 2020 583 711 719 10.1038/s41586-020-2077-3 32728246 PMC7410833
[8] Codutti L Leppek K Zálešák J A distinct, sequence-induced conformation is required for recognition of the constitutive decay element RNA by Roquin Structure 2015 23 1437 1447 10.1016/j.str.2015.06.001 26165594
[9] Janowski R Heinz GA Schlundt A Roquin recognizes a non-canonical hexaloop structure in the 3′-UTR of Ox40 Nat Commun 2016 7 11032 10.1038/ncomms11032 27010430 PMC5603727
[10] Binas O Tants J-N Peter SA Structural basis for the recognition of transiently structured AU-rich elements by Roquin Nucleic Acids Res 2020 48 7385 7403 10.1093/nar/gkaa427 32491174 PMC7367199
